# Heavy Metal Concentrations in the Groundwater of the Barcellona-Milazzo Plain (Italy): Contributions from Geogenic and Anthropogenic Sources

**DOI:** 10.3390/ijerph16020285

**Published:** 2019-01-21

**Authors:** Marianna Cangemi, Paolo Madonia, Ludovico Albano, Alessandro Bonfardeci, Maria Grazia Di Figlia, Roberto Maria Rosario Di Martino, Marco Nicolosi, Rocco Favara

**Affiliations:** Istituto Nazionale di Geofisica e Vulcanologia, Sezione di Palermo, via Ugo la Malfa 153, 90146 Palermo, Italy; paolo.madonia@ingv.it (P.M.); ludovicoalbano@libero.it (L.A.); alessandrobonfardeci@gmail.com (A.B.); difigliam@yahoo.com (M.G.D.F.); robertomr.dimartino@gmail.com (R.M.R.D.M.); marconicolosigeo@libero.it (M.N.); rocco.favara@ingv.it (R.F.)

**Keywords:** heavy elements, water quality, potential toxicity, human health, safe drinking-water, Maximum Admitted Concentrations, probability plots

## Abstract

We collected and analysed 58 samples of groundwater from wells in the Barcellona-Milazzo Plain, one of the most important coastal aquifers of Sicily (Italy), to determine major, minor, and trace element concentrations. In this area, geogenic and anthropogenic sources of heavy metals and other pollutants co-act, making the individuation of the main pollution sources difficult. Our work was aimed at the application of geostatistical criteria for discriminating between these pollution sources. We used probability plots for separating anomalous values from background concentrations, which were plotted on maps and related to possible sources of pollutants. Our results indicate that hydrothermal fluid circulation and the water–rock interaction of country rocks that host mineralized ore deposits generate a significant flux of heavy metals to groundwater, as well as anthropogenic sources like intense agriculture and industrial activities. In particular, NO_3_, F, and Ni exceed the Maximum Admitted Concentrations (MACs) established by the WHO and Italian legislation for drinking-water. The spatial distributions of geogenic and anthropogenic sources were so deeply interlocked that their separation was not easy, also employing geostatistical tools. This complex scenario makes the implementation of human health risk mitigation actions difficult, since the flow of pollutants is in many cases controlled by simple water–rock interaction processes.

## 1. Introduction

As stated by the World Health Organization (WHO), “access to safe drinking-water is essential to health, a basic human right, and a component of effective policy for health protection” [1]. 

Increasing global water demand, which has increased by a factor of six over the past 100 years [2] and is currently estimated to increase by about 1% per year, is a result of population growth, economic progress, and a change of the main uses of water [3]. For both industrial (20%, 75% of which is for energy production) and domestic (10%) usage, water demand will continue to increase, although agriculture water usage will remain the largest (70%) [3,4]. Moreover, this growing demand will increase especially in countries with developing or emerging economies [3]. 

Nowadays, about 3.6 billion people live in areas with water scarcity for at least one month per year [3]; moreover, the quality of water is deteriorating due to the impact of chemicals and pollutants, especially in lower-middle income countries, not only because of the growth of the population and the economy but also due to the lack of wastewater management systems [3].

These problems are particularly relevant for near-coastal areas, characterized by an average population density of 112 people km^−2^, above three times higher than the average global population density (44 people km^−2^) [5]. Among the chemical pollutants that significantly deteriorate water quality, metals and many other trace elements could impact human health. For this reason, governmental and private organisations have carried out risk assessments of chemicals for humans, and the WHO, since 1958, has published guideline values for safe drinking-water in the guide “International Standards for Drinking-Water”. 

The island of Sicily, located in the south Mediterranean, is the second-most populated region of Italy, with most of the people living in its coastal area. Groundwater, particularly in coastal aquifers, is by far the most important supply source for human consumption in Sicily. Due to the strategic importance of groundwater, the Sicilian Regional Government entrusted a study about the quality of groundwater for anthropic uses to Istituto Nazionale di Geofisica e Vulcanologia (INGV), Sezione di Palermo, released in 2007.

In this paper, with a specific focus on heavy metals, we present previously unpublished data acquired in that study related to the distribution and fate of major, minor, and trace elements in the main aquifer on the northern coast of Sicily (Italy). This area is densely populated and characterized by the presence of several different danger zones (industrial activities, intensive agriculture, and petroleum refineries). Moreover, the presence of mineralized deposits widely distributed inside the country rocks of the recharge area of this coastal aquifer constitutes a further relevant source for pollutants; thus, we focused our attention on the individuation of cases of potential toxicity for human health, discriminating between the geogenic and the anthropogenic source of the contaminants.

## 2. Study Area

**Figure 1:**
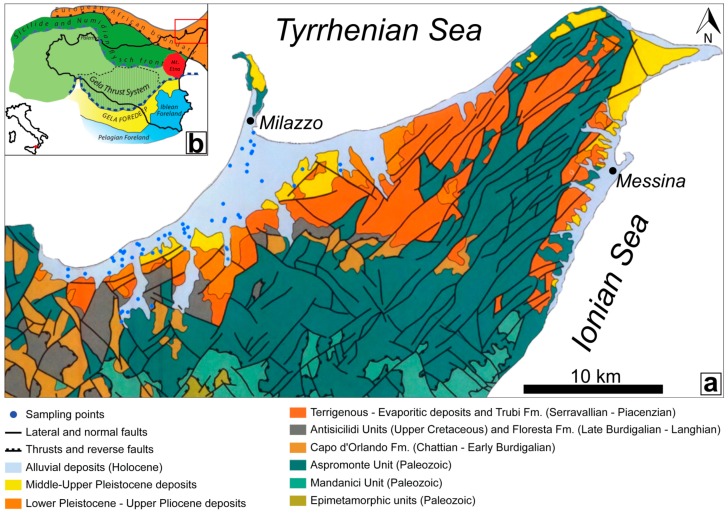
(**a**) Geological structure map of the easternmost part of Sicily (modified from [13]). On the map, the locations of the sampling points are shown (blue dots). (**b**) Main structural elements of the Sicilian Chain–Foredeep–Foreland System (modified from [7]).

The Peloritani Chain (PC) represents the easternmost and geometrically higher element of the Sicilian fold and thrust belt (Figure 1) [6,7,8]. 

It consists of a pile of superimposed tectonic units (nappes), derived from the delamination of part of the original south-eastern European margin and/or part of the Austroalpine-Alpine domain, consisting of a crystalline Paleozoic basement and related Meso-Cenozoic sedimentary units, from the late Oligocene–early Miocene [8,9,10,11,12]. 

In particular, the study area falls on the Tyrrhenian side of the Peloritani ridge, representing the main orographic component of north-eastern Sicily. The tectono-stratigraphic setting of this sector of the PC is characterized, from the bottom to the top [12,13,14], by the following:

- An extensive outcrop of the metamorphic rock succession belonging to the Aspromonte Mandanici, San Marco d’Alunzio, and Alì Units;
- A thick sin-orogenic deposits succession (conglomerates, turbiditic sandstones, and marly clays) belonging to the Capo d’Orlando Fm. (late Oligocene to early Miocene);
- The Antisicilide Unit (late Cretaceous to early Paleogene), composed of varicoloured clays with olistolithes of calcarenites and quartzarenites, that overthrusts the Capo d’Orlando Fm., is covered by the calcarenites of the Floresta Fm. (Burdigalian to Langhian);
- Post-orogenic units (middle Miocene to late Pleistocene deposits) that include terrigenous and evaporitic deposits.

Finally, the coastal sector is characterized by the large Barcellona–Milazzo alluvial plain. This plain, parallel to the coastline, is bordered by NW–SE and NE–SW fault systems [15,16]. This morpho-tectonic depression is filled by recent (Holocene) alluvial deposits, with a maximum thickness of 90 to 100 m [14,17], at the southern sector of the Milazzo peninsula.

The northern sector of the PC is characterized by the presence of metalliferous ore deposits [18] in marble layers and graphitic schists interbedded with ankerite, associated with fluorite, galena, and pyrite. Outcrops of sulphasalt-sulphide mineralisations of Cu-Sb-Ag-As, Ni, and Bi, with traces of Pb, Zn, W, and Au [19], are present above and laterally to the marble layers. These elements are found in metalliferous phases like pyrite, chalcopyrite, arsenopyrite, galena, sphalerite, pyrrhotite, sheelite, and tourmaline [19,20,21]. Moreover, a recent study [22] that investigated stream sediments highlighted anomalies of As, Sb, Zn, and Pb in the mineralized portion of the Mandanici Unit and at its tectonic contact with the Aspromonte Unit. 

A hydrothermal system is recognised in the studied area is located along the Tyrrhenian coast (Terme Vigliatore area) and is probably connected to the Aeolian–Tindari–Letojanni Fault System (ATLFS) [23]. The Barcellona–Milazzo Plain is characterized by intensive agriculture and by the presence of petrochemical plants.

## 3. Materials and Methods

Water samples taken in 58 wells drilled for human consumption and agricultural purposes were used to determine the dissolved major, minor, and trace elements. We used LD-PE (low-density polyethylene) bottles for major elements and HD-PE (high-density polyethylene) bottles for trace elements, which were pre-washed with ultrapure HNO_3_. Water samples were stored after filtration, using a 0.45 μm Millipore filter, and acidified to pH ca. 2 with ultrapure concentrated HNO_3_. Unacidified samples were stored for the analysis of anions. Aliquots, not filtered and not acidified, were used for alkalinity titration with HCl (0.1 N). The pH was measured with an 8102BN Ross combination pH electrode (ThermoFisher, Waltham, MA, USA), and the redox condition was tested using a Hamilton Oxitrode Pt 120 electrode (Hamilton, Manitowoc, WI, USA). Electric conductivity and temperature were measured using Thermo Orion instruments equipped with fabric electrodes (ThermoFisher, Waltham, MA, USA). Major concentrations were determined in the laboratories of INGV, Sezione di Palermo. Na, K, Mg, and Ca cations and Cl, F, NO_3_, NO_2_, and SO_4_ anions were determined by ionic chromatography using Dionex columns AS14 (ThermoFisher, Waltham, MA, USA) and CS12 (ThermoFisher, Waltham, MA, USA) for anions and cations, respectively. Ionic balance was computed for each sample taking into account the major species—all samples exhibited imbalance lower than 5%. Minor and trace element concentrations were determined at the University of Palermo (DiSTeM Department). The analyses were performed using an inductively coupled plasma-mass spectrometry (ICP-MS, Perkin Elmer Elan 6100 DCR-e, Perkin Elmer, Waltham, MA, USA), after using Re–Sc–Yas internal standards. Standard solutions were prepared with ultrapure deionized water, the ICP Multi Element Standard Solutions XXI CertiPUR, and the Sb CertiPUR standards (MERCK). The precision of the results was assessed by running triplicate analyses every 10th sample and fell within the range of 5–10%. Accuracy (±10%) was assessed by running SRM-1640 (groundwater) (NIST, Gaithersburg, MD, USA) and TMRAIN-95 (rain water) (NWRI, ON, Canada) reference standard materials. 

The dataset was processed using QGIS, version 3.2, in order to obtain the concentration maps of minor and trace elements over the target area. Each continuous raster surface of the maps models the spatial distribution of the specific element. The prediction surfaces were generated by the application of the ordinary kriging method, which generated a value at each point of the surface by the interpolation of a scattered set of points with z-values. The spherical model of autocorrelation was applied to the empirical semivariogram to obtain variograms and the covariance functions of the data.

## 4. Results

### 4.1. Chemico-Physical Parameters and Concentration of Major Elements 

Chemico-physical parameters measured in the field and the results of the chemical analyses are listed in Table 1. Values of pH, redox potential (Eh), electrical conductivity, and temperature range between 6.8 and 8.5, −29 and 253 mV, 390 and 1815 μS/cm, and 13.5 and 24.7 °C, respectively. These values are highly consistent with the interaction processes between groundwater and country rocks. Interaction with hydrothermal systems is evident in samples with higher temperatures.

**Figure 2:**
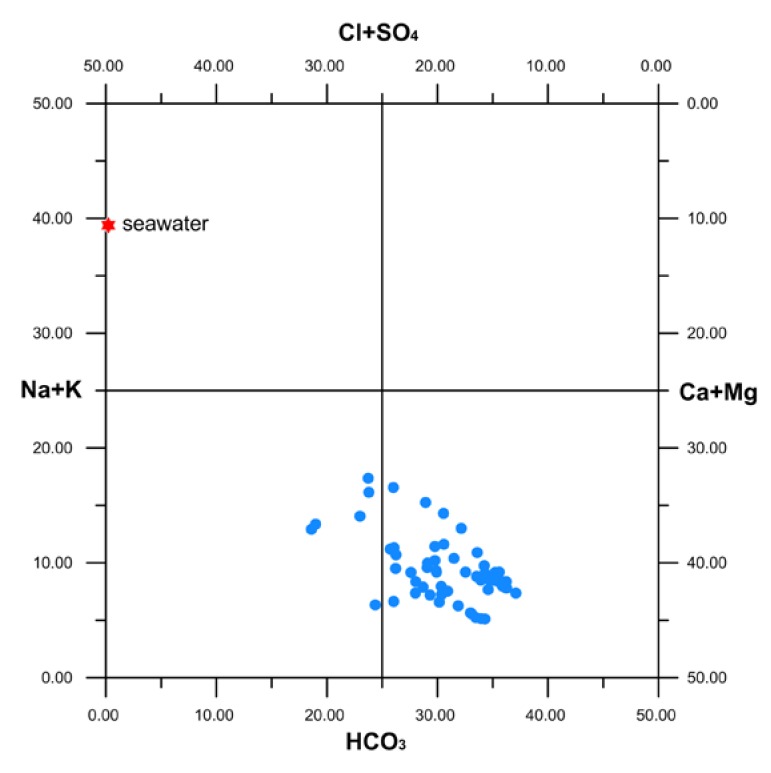
Langelier-Ludwig diagram [24] reporting the chemical data of groundwater (blue circles). The composition of Mediterranean seawater is also given for comparison.

**Figure 3:**
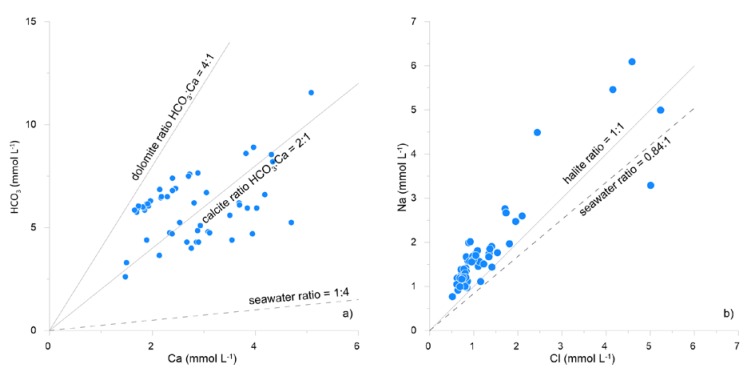
Scatter plots correlating the molar abundances of cation and anion species (data expressed in mmol L^−1^) in groundwater from the studied area, as (**a**) Ca vs. HCO_3_, and (**b**) Cl vs. Na. The grey lines represent the prevalent soluble mineralogical phases; the dashed line represents the seawater ratio.

Following the Langelier–Ludwig classification diagram [24] (Figure 2), the relative abundance of the main dissolved ions indicates that the studied groundwater mainly falls in the bicarbonate alkaline–earth-type quadrant, with a trend toward the Cl-sulphate alkaline-earth composition. The scatter plots reported in Figure 3 correlate the molar abundance of Ca vs. HCO_3_ and Na vs. Cl with those of seawater and of the main mineralogical phases from which these could derive. Comments are reported in the discussion section. 

### 4.2. Minor and Trace Element Concentrations

**Figure 4:**
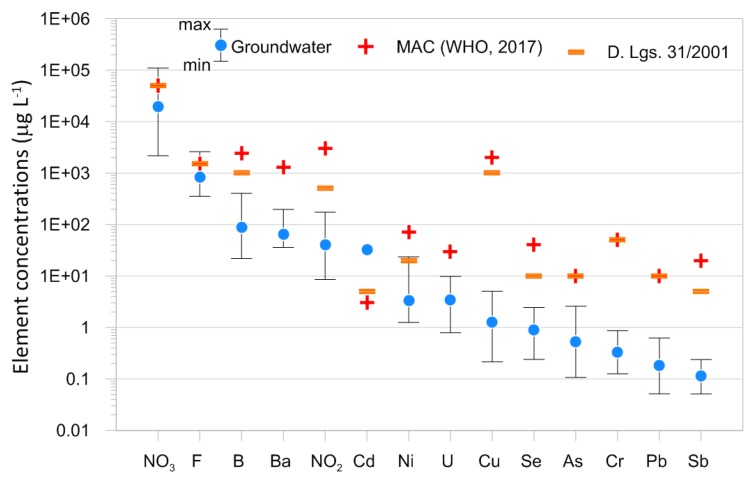
Mean values of trace element concentrations in groundwater (blue dots)—vertical black lines indicate related minima and maxima. Red crosses represent the Maximum Admitted Concentrations established by the WHO [1,25] for drinking-water, while orange dashes show the limits established by the Italian National Legislative Decree 31/2001 (2 February 2001) [26].

**Figure 5:**
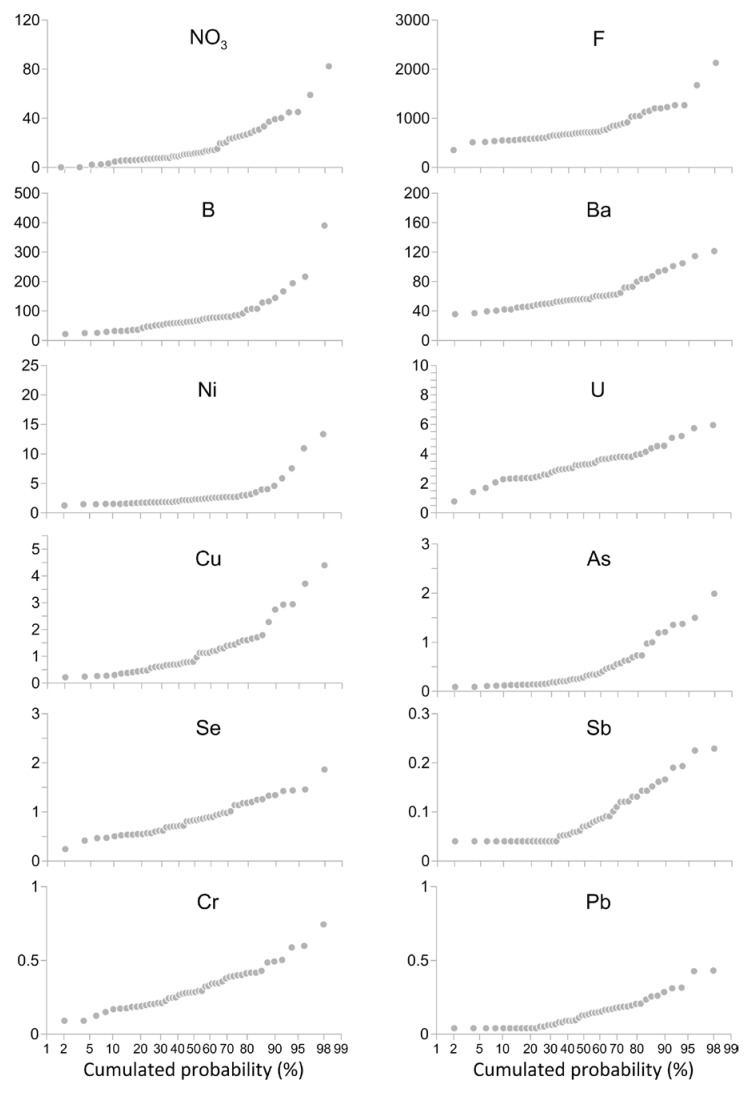
Probability plots of minor and trace elements. Concentrations are expressed in μg L^−1^, except for NO_3_, which is expressed in mg L^−1^.

Minor and trace element concentrations range from 0.05 μg L^−1^ for elements like Sb and Pb up to 110 mg L^−1^ for NO_3_ (Table 1). Their distribution in groundwater is summarized in Figure 4 and compared to the Maximum Admitted Concentrations (MACs) indicated by the WHO [25] and the Italian National Legislative Decree (D. Lgs.) 31/2001 [26]. Probability plots (Figure 5) were drafted in order to discriminate the background values and different families of data. The probability plots express the probability of cumulative frequencies of the measured concentrations of each trace element. In these plots, each log-normal population of data shows as a straight line, whereas an inflection point divides two populations [27].

**Figure 6:**
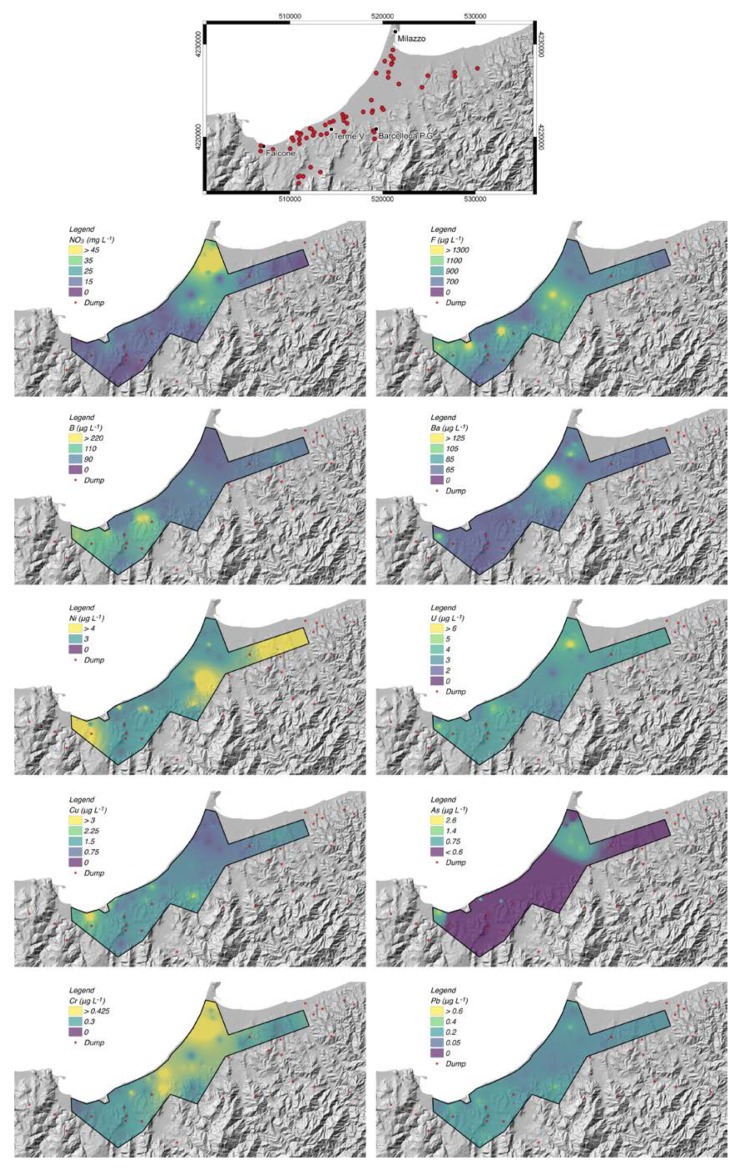
Spatial distribution of selected chemicals. The map at the top shows the locations of the sampling sites and the coordinates of the studied area. Base map TINITALY (Triangular Irregular Network of Italy) DEM [28,29,30].

Based on the results of the probability plots, we mapped the spatial distribution of concentrations of selected chemicals in function of the inflection points on the probability plot curves—the results are shown in Figure 6. We only mapped chemicals showing spatial anomalies emerging from the background, thus excluding all those uniformly distributed. The distribution of concentrations exhibits a general spatial coherence, with relative high concentration areas often coinciding for different chemical species and, in particular, moving from SW to NE: i) the south of the city of Falcone; ii) the Terme Vigliatore area; iii) and the Milazzo Plain.

## 5. Discussion

Following the indications of the Langelier–Ludwig diagram (Figure 2), the general water chemistry reflects the abundance of cations and anions in the lithological matrix, which is dominated by the dissolution of bicarbonate and the hydrolysis of aluminosilicate minerals. Further details are given by the scatter plots in Figure 3. The Ca vs. HCO_3_ diagram indicates that calcium is mainly derived from the dissolution of limestones and dolostones, while a minor contribution comes from it mixing with variable amounts of seawater. The leaching of Na-aluminosilicates, like albite, and/or the ion-exchange reactions between water and clay minerals [31] can explain the Na excess with respect to both seawater and halite dissolution evidenced in the Cl vs. Na diagram. None of the major ions found in the aquifer of the Barcellona–Milazzo Plain area exceed the MACs indicated by the WHO [1,25] and/or the D. Lgs. 31/2001 [26].

On the contrary, the concentrations of minor and trace elements above the MACs were found for NO_3_ (3 samples), F (3 samples), and Ni (1 sample) (Figure 4). Other elements not exceeding the related MACs but showing concentrations significantly higher than the backgrounds evidenced in Figure 5 are As, B, Ba, Cr, Cu, and U.

Concentration maps of these chemicals were plotted in order to evaluate if high concentration values were in proximity to possible geogenic and/or anthropic sources. Moving from SW to NE, three main high concentration areas can be individuated: (i) south of the city of Falcone, with enrichments of F, Cu, Ba, Ni, As, and secondarily U, (ii) the Terme Vigliatore area, characterized by high values of F, B, U, Cu, and, to a lesser extent, Ni and As, and (iii) in a NNE–SSW zone between Milazzo and Barcellona, with enrichments in NO_3_, As, U, Cr, F, and, to a lesser extent, Cr, Ba, and Pb.

A first possible source for many of these elements, like B, F, As, Pb, and Cu, can be found in the rising hydrothermal fluids enriched in sulphide phases. In particular, the Terme Vigliatore area is characterized by geothermal springs and gas vents, which are the surface expression of the migration of sub-crustal fluids along a complex network of tectonic structures [32]. In this scenario, B acts as a carrier for metals [33]. Arsenic is predominantly transported under the form of neutral aqueous species and oxy-anions. It is highly mobile in geothermal systems and is a good indicator of the leaching degree of its host rocks, due to progressive water–rock interaction processes [34]. Geothermal waters, once in contact with the atmosphere, produce sulphide precipitates of antimony and arsenic with the co-precipitation of mercury [35]. The elements that are found in significant concentrations in groundwater are the same as those recognized in the mineralized portion of the Peloritani mountain range [18,19,20] and are linked to the synsedimentary deposition of metalliferous ore deposits in euxinic basins, associated with sulphides [18], and are successively metamorphosed. However, anthropic sources cannot be ruled out. In the Milazzo area, industrial activities like refineries and electrical plants emit pollutants into the atmosphere and water [36], because oil combustion and fluidized-bed catalytic cracking produce a flux of elements, like Ni and Cr in refineries and Pb, Co, and Cu in electrical plants. In particular, the relative high concentration of Ni found in the sampling points close to Milazzo could be due to the wet deposition of atmospheric particulates emitted by the refineries’ chimneys. Moreover, other activities like road traffic or illegal waste combustion also release toxic substances [36]. The Barcellona–Milazzo Plain is characterized by intense agriculture and industrial activities where volatile organic compounds like tetrachloroethylene (PCE) and chloroforms (TMC), used as fumigants and insecticides, have been found in concentrations exceeding the guideline values proposed by the Italian legislation [37]. In this area, the highest NO_3_ concentrations have been found. For example, both NO_3_^−^ and F, can reach both surface water and groundwater as a consequence of intensive agricultural activity, from wastewater disposal, and in particular for NO_3_^−^ from the oxidation of nitrogenous waste products in human and other animal excreta, including septic tanks [1,25]. Fluorine is contained in vegetation and is absorbed from the soil and water [1,25], whereas Cd is used in the steel industry, in plastics, and in batteries, and is released into the environment through wastewater [1,25].

NO_3_, F, and Ni exceeded their MACs in the studied area. The adverse effects of these elements on human health have been widely investigated by the WHO [1,25]. In particular, nitrates in drinking-water may be a risk factor for methaemoglobinaemia in infants, fostered by the presence of simultaneous gastrointestinal infections, which increase endogenous nitrite formation [1,25]. Epidemiological studies have been carried out on the association of nitrate intake primarily with gastric cancers, which appears to inhibit iodine uptake and has the potential for an adverse effect on the thyroid [1]. Dental fluorosis is connected to an overexposure (>1.5 mg L^−1^) of fluoride during childhood, when teeth develop [38]. Higher concentrations of F (3–6 mg per litre) lead to skeletal fluorosis [38]. The International Agency for Research on Cancer (IARC) has classified Ni and their compounds as probably carcinogenic for humans [1,25]. In humans, acute oral Ni poisoning results primarily in haemorrhagic gastritis and colitis, with ultimate damage to the kidney [1,25].

## 6. Conclusions

The study area is a particularly complex scenario where geogenic and anthropogenic sources of minor and trace elements and other pollutants, potentially toxic for humans, co-act.

From the geogenic point of view, toxic chemicals can be derived from hydrothermal fluids, mineralized areas, and/or rock–water interactions. The petrographic nature of country rocks is characterized by the presence of metalliferous ore deposits, which can release heavy elements into groundwater. Possible anthropogenic sources are industrial activity, like refineries, electrical plants, or intensive agriculture. Chemical species like NO_3_, F, and Ni exceed the MACs established by the WHO and the Italian National Legislative Decree 31/2001 for drinking-water.

In this area, it is not always correct to use the standardized threshold values for heavy metal concentrations, whereas local backgrounds for selected elements should be carefully identified with the aim to design a risk analysis for the pollutants. The spatial distribution of these pollutants does not allow for the separation of the two different possible sources, because the anthropic activities potentially able to generate a flux of hazardous chemicals are widely distributed in the studied area. This scenario implies difficulties in implementing any risk mitigation actions, because, even in the absence of anthropogenic pollution sources, the simple water–rock interaction processes can contaminate aquifers exploited for human consumption.

## Figures and Tables

**Table 1:** Mean, median, minimum, maximum, and 1st and 3rd quartile values, standard deviation (σ), and detection limits of temperature (°C) for Eh (mV), pH, electric conductivity (EC, μS cm^−1^), and major (mg L^−1^), minor and trace element (μg L^−1^) concentrations in groundwater. The letter n in brackets indicates the number of samples with concentrations over the instrumental detection limit. Maximum Admitted Concentrations, established by the WHO [25], and the Italian National Legislative Decree (31/2001) [26] limits are also reported. n.a. means not applicable.

	Mean	Median	Minimum	Maximum	1st Quartile	3rd Quartile	σ	Detection Limits	WHO Limits	D. Lgs. 31/2001 Limits
T (°C)	17.5	17.1	15	24.7	16.4	18.3	1.6	n.a.	n.a.	n.a.
Eh (mV)	144	168.5	-29	253	98.75	189	64.7	n.a.	n.a.	n.a.
pH	7.28	7.26	6.81	8.49	7.15	7.39	0.2	n.a.	n.a.	n.a.
EC (20 °C, uS cm^−1^)	922	876	502	1815	766	985	262	n.a.	n.a.	n.a.
Na (mg L^−1^)	42.6	35.2	17.6	140.0	27.2	42.3	27.3		n.a.	n.a.
K (mg L ^−1^)	5.99	5.67	2.96	13.6	4.77	6.46	2.1		n.a.	n.a.
Ca (mg L^−1^)	111	105	55	217	77	136	38.5		n.a.	n.a.
Mg (mg L^−1^)	33.5	35.9	10.7	68.3	18.0	44.7	16.6		n.a.	n.a.
Cl (mg L^−1^)	49.0	32.4	18.3	236.8	27.4	48.1	44.4		n.a.	n.a.
SO_4_ (mg L^−1^)	124	108	64.8	307	85.3	147	49.5		n.a.	n.a.
HCO_3_ (mg L^−1^)	368	368	159	705	294	415	94.9		n.a.	n.a.
NO_3_ (mg L^−1^) (n=58)	19.5	11.8	2.18	110	7.55	25.4	19.7	<1	n.a.	50
F (μg L^−1^) (n = 52)	847	711	354	2596	604	943	401		1500	1500
Sb (μg L^−1^) (n = 49)	0.115	0.101	0.051	0.238	0.072	0.143	0.1	<0.05	20	5
As (μg L^−1^) (n = 49)	0.53	0.328	0.107	2.58	0.175	0.664	0.5	<0.1	10	10
Ba (μg L^−1^) (n = 49)	65.4	56.2	36	196.4	49.6	72.4	27.8	<1	1300	n.a.
B (μg L^−1^) (n = 49)	88.0	67.2	21.7	403.5	51.6	86.2	76.4	<0.1	2400	1000
Cr (μg L^−1^) (n = 49)	0.332	0.294	0.1255	0.87	0.212	0.401	0.2	<0.1	50	50
Ni (μg L^−1^) (n = 49)	3.31	2.32	1.25	23.6	1.79	2.75	3.7	<0.1	70	20
Pb (μg L^−1^) (n = 49)	0.182	0.160	0.051	0.624	0.092	0.207	0.1	<0.05	10	10
Cu (μg L^−1^) (n = 49)	1.25	0.965	0.215	5.04	0.604	1.52	1.06	<0.1	2000	1000
Se (μg L^−1^) (n = 49)	0.902	0.835	0.24	2.44	0.596	1.14	0.4	<0.1	40	10
U (μg L^−1^) (n = 49)	3.44	3.3	0.79	9.84	2.6	3.8	1.4	<0.1	30	n.a.
